# Neuroprotective effects of apigenin against inflammation, neuronal excitability and apoptosis in an induced pluripotent stem cell model of Alzheimer’s disease

**DOI:** 10.1038/srep31450

**Published:** 2016-08-12

**Authors:** Rachelle Balez, Nicole Steiner, Martin Engel, Sonia Sanz Muñoz, Jeremy Stephen Lum, Yizhen Wu, Dadong Wang, Pascal Vallotton, Perminder Sachdev, Michael O’Connor, Kuldip Sidhu, Gerald Münch, Lezanne Ooi

**Affiliations:** 1 Illawarra Health and Medical Research Institute, School of Biological Sciences, University of Wollongong, Wollongong, NSW, 2522, Australia; 2 School of Medicine, Western Sydney University, Locked bag 1797, Penrith, NSW, Australia; 3 Illawarra Health and Medical Research Institute, School of Medicine, University of Wollongong, Wollongong, NSW, 2522, Australia; 4 CSIRO Informatics and Statistics, Locked Bag 17, North Ryde, NSW 1670, Australia; 5 Centre for Healthy Brain Ageing School of Medicine, University of New South Wales, High Street, Kensington,. NSW, 2052, Australia; 6 Molecular Medicine Research Group, Western Sydney University, Locked bag 1797, Penrith, NSW, Australia; 7 Centre of Complementary Medicine Research (CompleMed), Western Sydney University, Locked bag 1797, Penrith, NSW, Australia

## Abstract

Alzheimer’s disease (AD) is one of the most prevalent neurodegenerative diseases, yet current therapeutic treatments are inadequate due to a complex disease pathogenesis. The plant polyphenol apigenin has been shown to have anti-inflammatory and neuroprotective properties in a number of cell and animal models; however a comprehensive assessment has not been performed in a human model of AD. Here we have used a human induced pluripotent stem cell (iPSC) model of familial and sporadic AD, in addition to healthy controls, to assess the neuroprotective activity of apigenin. The iPSC-derived AD neurons demonstrated a hyper-excitable calcium signalling phenotype, elevated levels of nitrite, increased cytotoxicity and apoptosis, reduced neurite length and increased susceptibility to inflammatory stress challenge from activated murine microglia, in comparison to control neurons. We identified that apigenin has potent anti-inflammatory properties with the ability to protect neurites and cell viability by promoting a global down-regulation of cytokine and nitric oxide (NO) release in inflammatory cells. In addition, we show that apigenin is able to protect iPSC-derived AD neurons via multiple means by reducing the frequency of spontaneous Ca^2+^ signals and significantly reducing caspase-3/7 mediated apoptosis. These data demonstrate the broad neuroprotective action of apigenin against AD pathogenesis in a human disease model.

Alzheimer’s disease (AD) is a neurodegenerative disorder involving the progressive loss of neurons in the brain^1^. The precise etiology is unknown for the majority of AD patients, who suffer from sporadic or late-onset AD; however, advanced age and inheritance of the ε4 allele of the apolipoprotein E gene are significant risk factors^2^. For a subset of patients, who suffer from the familial or early-onset form of the disease, specific genetic mutations have been identified. These include mutations in the presenilin-1 or -2 genes (*PSEN1*, *PSEN2*), as well as the amyloid precursor protein (encoded by *APP*)^3^,^4^. These mutations directly contribute to the accumulation of one of the pathogenic protein hallmarks of AD, β-amyloid (Aβ), which aggregates to form extracellular plaques that can disrupt a multitude of cellular functions^4^. For example, amyloid peptide accumulation has been reported to cause disruptions to the tightly regulated neuronal calcium (Ca^2+^) signalling systems during the progression of AD, affecting synaptic plasticity, memory and learning^5^, and contributing to a wide range of downstream pathogenic effects, such as the generation of reactive oxygen and nitrogen species^6^. Furthermore, accumulation of Aβ has been shown to cause inflammation, leading to the activation of microglia around Aβ plaques^7^. These activated microglia likely contribute to the increased levels of cytokines and chemokines, including interleukin-1 (IL-1), IL-6, and tumour necrosis factor α (TNF-α), observed in AD brains^7^,^8^. The second major pathogenic hallmark of AD is the accumulation of intracellular neurofibrillary tangles, which form when the microtubule associated protein tau becomes hyperphosphorylated, destabilising microtubules^9^. It has been reported that inflammation and reactive nitrogen species, which can cause the nitration of tau tyrosine residues, can further contribute to the aggregation of tau^10^. The combined action of these pathogenic factors likely contributes to the loss of synapses, neurite retraction and ultimately the death of neurons.

The rate of neurodegeneration in AD, from the initial pathogenic changes in neuronal signalling, to the presentation of clinical symptoms and the subsequent cognitive decline, differs between individuals^1^. This has led to suggestions that both genetic and environmental factors play a significant role in the progression of this neurodegenerative disease^1^. This concept is supported by epidemiological studies that have positively correlated environmental and behavioral aspects, such as diet and lifestyle, with incidents of AD and other forms of dementia (reviewed in refs 11,12). The combination of these factors indicates that the most effective treatments for AD might be pre-emptive, targeting the prevention or delay of onset rather than attempting to reverse pathogenic mechanisms once symptoms have developed.

Research has indicated that dietary based neuroprotective, anti-inflammatory and anti-oxidant agents may successfully reduce the risk or delay the onset or progression of AD^13^. Indeed non-steroidal anti-inflammatory drugs (NSAIDs) decrease the risk of AD^14^, though their serious side effects prevent long-term use^15^. Our previous studies have used macrophage and microglial cell lines to systematically screen compounds for their anti-inflammatory activity^16^ and cytoprotective properties in inflammation and oxidative stress-induced cell death in astrocyte^17^ and neuronal cell lines^18^. From these studies we have identified the polyphenolic compound apigenin, which is present in celery, parsley and artichoke, as able to reduce both tumour necrosis factor-α (TNFα) and NO release from RAW264.7 macrophages, following activation with lipopolysaccharide^19^, however in these studies only a limited cytokine repertoire was assessed. These data suggest that apigenin could possess neuroprotective properties in the pro-inflammatory setting of AD.

Several groups have reported the anti-inflammatory action of apigenin in a number of human and animal inflammatory cell lines and animal models^20^,^21^,^22^. Furthermore, analysis of the neuroprotective potential of apigenin in a double transgenic mouse model of AD (APP/PS1) indicated that apigenin could ameliorate AD-associated memory impairment, reduce the Aβ plaque burden and inhibit oxidative stress^23^. Several groups have reported anti-apoptotic effects of apigenin in murine HT22^24^ and human SH-SH5Y cell lines^25^ and shown that apigenin can reduce glutamate-induced Ca^2+^ signalling in murine cortical neurons^26^. Collectively, these results indicate that apigenin may have potent neuroprotective properties. However its contribution to neuroprotective mechanisms in human AD neurons is currently unknown.

Studying the temporal and cellular responses of neurons from AD patients to novel therapeutics has been difficult. A primary setback in the development of effective treatments for AD is the disparity between the outcomes in multiple cell and animal based models in comparison to clinical trials^27^. These models often over-express the pathogenic protein hallmarks of AD to induce the disease phenotype, while failing to fully recapitulate vital aspects of pathogenesis, such as gross synaptic loss and neuronal death^28^,^29^. The advent of induced pluripotent stem cell (iPSC) models has provided the opportunity to interrogate AD pathogenesis in human disease-specific neurons^30^ that express endogenous levels of disease-relevant proteins^31^.

We carried out a comprehensive and complementary analysis of the anti-inflammatory and neuroprotective activity of apigenin in human iPSC-derived neurons from familial, sporadic AD or healthy individuals. The iPSC-derived AD neurons used in the present study have shortened neurites and reduced neuronal viability, as well as elevated levels of nitrite and apoptosis. In addition, we identified a hyper-excitable Ca^2+^ signalling phenotype in the iPSC-derived AD neurons, which demonstrated spontaneous Ca^2+^ signals in the absence of stimulus. By developing a co-culture model using iPSC-derived neurons together with activated murine macrophages, we show that inflammation and oxidative stress lead to reduced neurite length and neuronal viability within AD patient-derived neurons, and that this effect can be prevented with apigenin. We show that apigenin is able to protect neurons from apoptosis (but not cytotoxicity), as well as reduce the hyper-excitable Ca^2+^ signalling phenotype in AD neurons and glutamate signalling in healthy neurons. Together our data highlight a broad neuroprotective action of apigenin via multiple mechanisms against AD pathogenesis in a human disease model.

## Results

### Apigenin protects familial AD neurons from inflammatory stress

Inflammatory signalling molecules from activated glia can cause neuronal death^7^,^8^,^18^. To identify whether apigenin could protect against inflammation-induced neuronal death in our cellular model, we first carried out a comprehensive analysis of the anti-inflammatory activity of apigenin in the murine macrophage cell line RAW264.7, microglial cell line C8-B4, and primary microglia, all of which are commonly used as cellular models of inflammation^18^. In each of these cell types apigenin is able to down-regulate NO and a range of pro-inflammatory cytokines with IC_50_ values between 10 and 100 μM (Supp. Tables 1–3), consistent with previous studies^20^,^21^.

**Figure 1:**
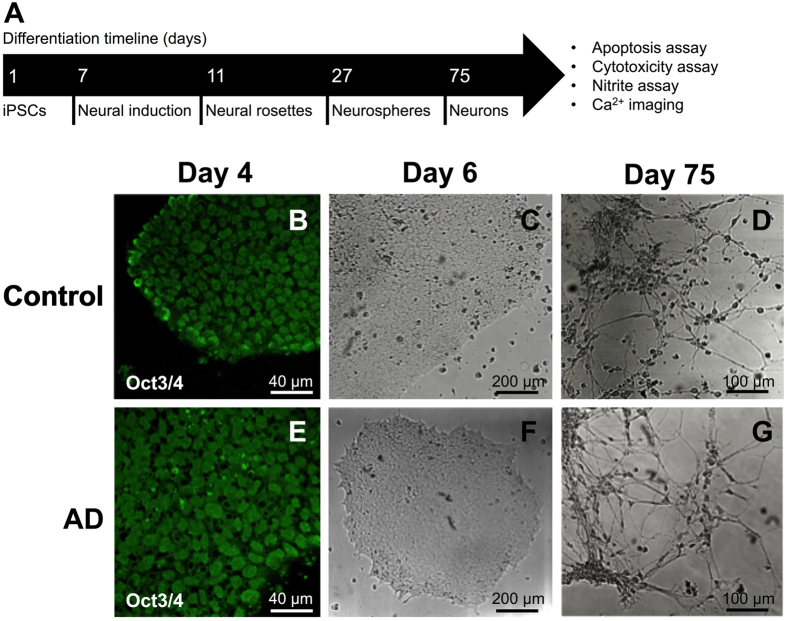
Neuronal differentiation timeline (**A**) with (**B**–**D**) control and (**E**–**G**) AD derived iPSC cells. (**B**,**E**) immunostaining of pluripotent stem cell marker Oct3/4, (**C**,**F**) brightfield images of stem cell colonies and (**D**,**G**), cells after differentiation into neurons.

**Figure 2:**
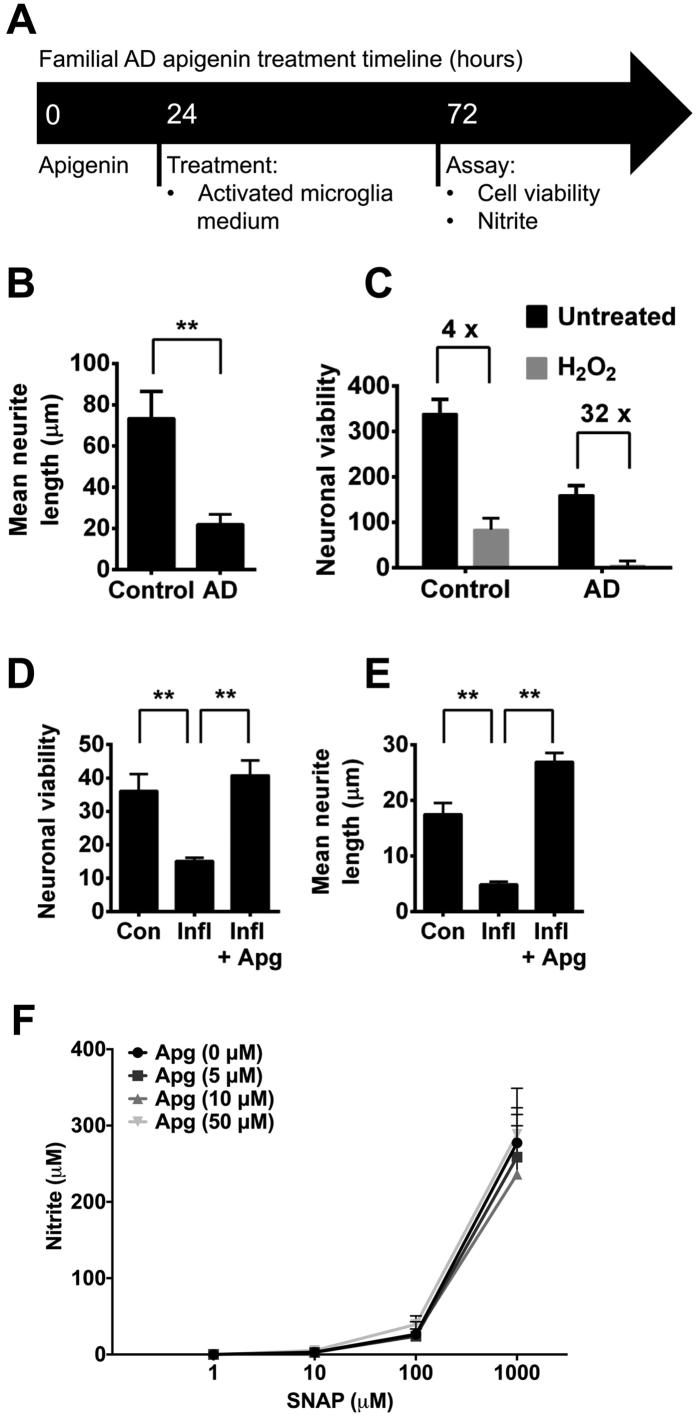
Apigenin treatment regime for familial AD iPSC-derived neurons (**A**). Neurons were generated from iPSCs from a familial AD patient carrying a *PSEN1* (P117R) mutation or an age-matched control. (**B**) Length of neurites from familial AD or control neurons was measured using HCA-vision software. All neurites were measured in >10 images per experiment, n = 3. **indicates significant difference (p ≤ 0.01), paired t-test. (**C**) Neurons were treated with vehicle control or 100 μM H_2_O_2_ for 24 h and viability of AD or control neurons was measured. (**D**) Neuronal viability and (**E**) neurite length were measured in AD neurons or those cultured in media taken from activated microglia under inflammatory conditions (Infl; microglia activated with LPS (50 μg/ml) and IFN-γ (20 U/ml) for 48 h ± 50 μM Apigenin (Apg; 24 hour pre-incubation). (**F**) Nitrite formation in cell culture medium in the absence of cells treated with SNAP (0, 1, 10, 100, 1000 μM) and apigenin (0, 5, 10, 50 μM). Data shown are mean ± standard error of the mean from 3 independent experiments.

In order to determine whether these anti-inflammatory effects would lead to neuroprotection in a human neuronal model of AD, we first generated iPSCs from a patient with familial AD, bearing a mutation in *PSEN1* (P117R), and an age-matched control. This mutation has a symptomatic age of onset around 35 years of age^32^ and leads to an aggressive phenotype that we hypothesised would generate a robust cellular AD model. As shown previously^30^,^33^, the iPSCs expressed Oct3/4 and grew in colonies (Fig. 1). The iPSCs could be differentiated into neurons with extended neurites (Fig. 1). Western blot analysis of familial AD and control neurons at 35 days of differentiation identified the expression of synapse markers PSD-95 and Synapsin I, both of which are required for formation and maturation of synapses^34^ (Supp. Fig. 1), in addition to the dendrite marker MAP2 (Supp. Fig. 1). However, after 75 days of differentiation, the iPSC-derived neurons from the familial AD patient displayed reduced neurite length, compared to control neurons (Fig. 2A,B).

Using this disease model, treatment with H_2_O_2_ (100 μM) for 24 h to induce oxidative stress led to neuronal death, causing a greater reduction in neuronal viability in neurons from the familial AD patient (32 fold) than the control (4 fold; Fig. 2C; p < 0.01).

Our current results and previously published work^16^ show that inflammatory activation of microglia and macrophages increases NO and cytokine release. As inflammation and elevated levels of nitrosative and oxidative stress are associated with neurodegeneration during AD^7^,^35^, we then investigated the effect of conditioned media from activated primary microglia on AD neurons in combination with the neuroprotective effect of apigenin. Exposure of the iPSC-derived AD neurons to conditioned medium for 48 h led to a reduction in both neuronal viability (Fig. 2D) and neurite length (Fig. 2E). We used a concentration of 50 μM apigenin, in line with our previous IC_50_ values, to test the neuroprotective activity of apigenin. Pre-incubation (24 h) with apigenin (50 μM) protected against both neuronal death and neurite shortening in AD neurons (Fig. 2E,F).

To investigate whether the neuroprotective mechanism of apigenin was due to its ability to scavenge the high levels of NO produced by activated glial cells, nitrite formation was measured by Griess assay following treatment with the NO donor, S-Nitroso-N-acetyl-DL-penicillamine (SNAP), in cell medium in the absence of cells. There was no significant difference in nitrite levels across all concentrations of apigenin and SNAP treatments (Fig. 2F), suggesting that apigenin does not directly scavenge NO in the cell medium.

Together, these data demonstrate reduced neurite length and cell viability in familial AD neurons, which was exacerbated by exposure to oxidative and inflammatory stresses. Treatment with apigenin protected neurons against the inflammatory stress induced neurite retraction and death.

### Apigenin reduces apoptosis in sporadic AD and control neurons

**Figure 3:**
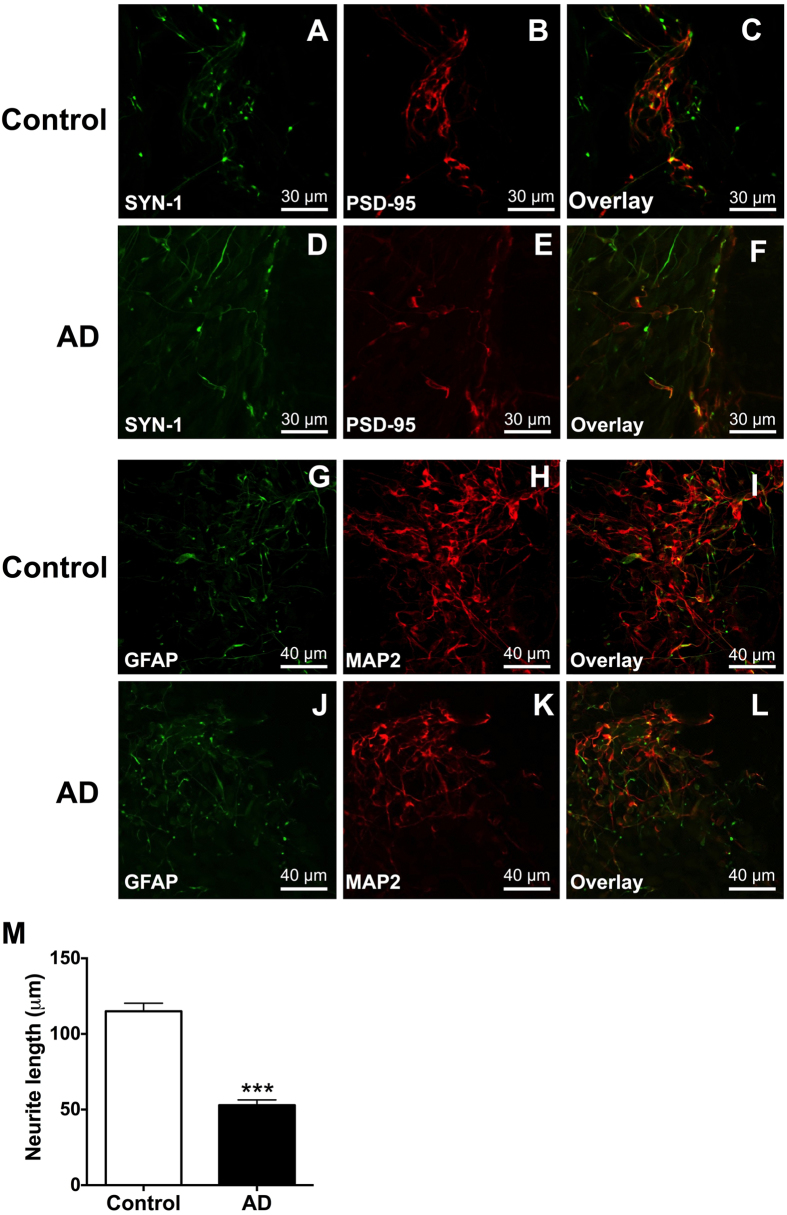
Neurons were generated from iPSCs from a sporadic AD patient or an age-matched control. Immunostaining of neurons in culture with (**A**,**D**) Synapsin I (SYN-1) and (**B**,**E**) PSD-95, (**C**,**F**) overlay or (**G**,**J**) GFAP and (**H**,**K**) MAP2, (**I**,**L**) overlay. (**M**) Length of neurites from sporadic AD or control neurons presented as mean ± SEM of three independent experiments. ***Indicates significant difference (p ≤ 0.001), paired t-test.

Familial AD neurons provide a good model to understand early-onset AD, however since sporadic, late-onset AD is the most common form of the disease and is difficult to model using animals^36^, we focused the remainder of our study on sporadic AD neurons. In order to further interrogate the potential neuroprotective effects of apigenin, we differentiated iPSCs derived from a sporadic AD patient and an age and gender matched control. As shown previously^25^, these iPSCs form stem cell colonies, express markers of pluripotency and form teratomas following implantation into immune-compromised mice (data not shown). For the present study these sporadic AD and control iPSCs were differentiated into neurons with extended neurites, with immunocytochemistry demonstrating the expression of the synaptic maturation protein Synapsin I, the post-synaptic marker PSD-95 (Fig. 3A–F) and the neuronal microtubule marker MAP2 (Fig. 3G–L), as well as GFAP-positive neural precursors/cells (Fig. 3G–L). The iPSC-derived neurons from the sporadic AD patient released 2.6 fold higher levels of Aβ_42_ compared to neurons from the control patient (t_(4)_ = 3.426, p < 0.05), however there was no significant difference in Aβ_40_ release (Supp. Fig. 2), which is in accordance with the results of several other studies^31^,^37^,^38^.

As the loss of synapses from neurite retraction is one of the strongest pathological correlates with cognitive decline in AD^39^, we assessed neurite length. Similar to familial AD iPSC-derived neurons, the neurites of sporadic AD neurons were 54% shorter at 52 days of differentiation, in comparison to control neurons (t_(39)_ = 8.15, p < 0.001; Fig. 3M). This result is further supported by western blot analysis of iPSC-derived neurons, which identified a significant decrease in the dendrite marker and microtubule associated protein, MAP-2C, in sporadic AD neurons in comparison to the control, at 52 days of differentiation (t_(12)_ =  2.276, p < 0.05; Supp. Fig 3). However, there was no difference in the expression levels of the neuronal markers, neuron specific enolase (NSE) and NeuN, nor synaptic marker, synapsin-1, or axonal marker, neurofilament heavy chain, between control and sporadic AD neurons, suggesting there was no difference in the number of neurons present in the cultures.

**Figure 4:**
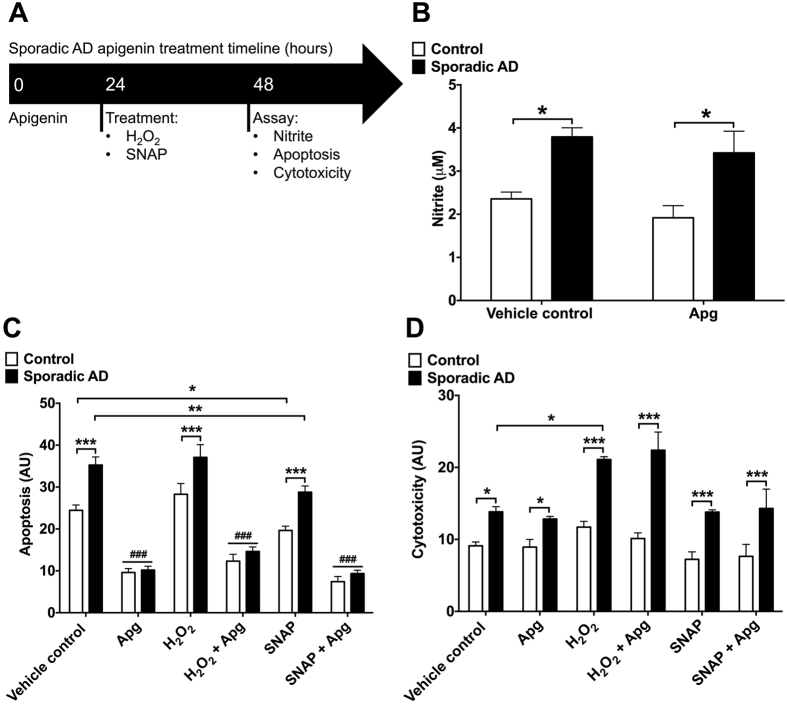
(**A**) Apigenin treatment regime for sporadic AD and control neurons. (**B**) Nitrite concentration in the cell medium was measured by Griess assay in the presence of vehicle control or apigenin (50 μM treatment for 24 h). (**C**) Apoptosis and (**D**) cytotoxicity were simultaneously measured using an Apotox Glo kit. Control or AD neurons were treated with vehicle control or apigenin (50 μM), H_2_O_2_ (300 μM) or SNAP (10 μM) for 24 h. Data are presented as mean ± SEM of three independent experiments. Significant differences from matched treatment are indicated by *p ≤ 0.05 and ***p ≤ 0.001, with significant difference from control treatment indicated by ^###^p ≤ 0.001 using two-way ANOVA with Tukey’s multiple comparisons test.

Central to neurodegeneration is the loss of neurons by cell death pathways, due to multiple contributing factors in AD, potentially including oxidative and nitrosative stress^1^. We therefore used our cellular model to assess the effects of apigenin on the levels of cytotoxicity and apoptosis. In the brain, NO can be both neuroprotective and neurotoxic, depending on the local concentration; we have previously shown that exogenous application of high levels of NO is neurotoxic to cell lines like Neuro2a^19^, however at low concentrations NO can be neuroprotective (reviewed in ref. 35). Neurons were differentiated for 75 days, prior to 50 μM apigenin or vehicle control pre-treatment (24 h), followed by 300 μM H_2_O_2_ or 10 μM SNAP treatment for 24 h prior to analysis (Fig. 4A). Sporadic AD neurons had a significantly higher basal level of nitrite (F(1, 8) = 64.69, p < 0.001), used as a downstream marker of NO, present in cell culture medium, compared to control neurons (p < 0.05, Fig. 4B). Pre-treatment of the cells for 24 h with 50 μM apigenin caused a small but non-significant reduction in basal nitrite levels (F(1, 8) = 4.89, n.s.), in comparison to vehicle treatment in both healthy control and sporadic AD cells. This is consistent with our previous results that suggest that apigenin does not scavenge NO in cell culture medium (Fig. 2). The sporadic AD neurons exhibited a significantly higher level of caspase-3/7 activation (F(5, 24) = 11.16) than healthy control neurons, used as a marker of apoptosis (p < 0.001, Fig. 4C,D). Pre-treatment with apigenin (50 μM) for 24 h significantly reduced caspase-3/7 activity levels of sporadic AD neurons (p < 0.001) to the level of control neurons (Fig. 4C). Consistent with our previous experiments showing that neurons from a familial AD patient displayed reduced viability following H_2_O_2_ treatment (Fig. 2), levels of cytotoxicity (F(5, 24) = 8.26, p < 0.001) were higher in the sporadic AD neurons than controls and cytotoxicity increased following treatment with H_2_O_2_, promoting oxidative stress (p < 0.001; Fig. 4D). Although apigenin protected both sporadic AD and control neurons against apoptosis it had no effect on cytotoxicity (Fig. 4D).

To establish the effect of exogenous NO on these neurons, the cells were treated with the NO donor SNAP (10 μM). Treatment with SNAP caused a small but significant reduction in apoptosis (F(5, 24) = 249.5, p < 0.001) for both sporadic AD (p < 0.01) and control neurons (p < 0.05), but did not affect cytotoxicity (Fig. 4C,D). Collectively, these data identify significantly increased levels of apoptosis, cytotoxicity and nitrite, in conjunction with reduced neurites in sporadic AD neurons and that apigenin and low levels of exogenous NO are able to reduce apoptosis.

### Apigenin treatment limits apoptosis in sporadic AD and control neurons

**Figure 5:**
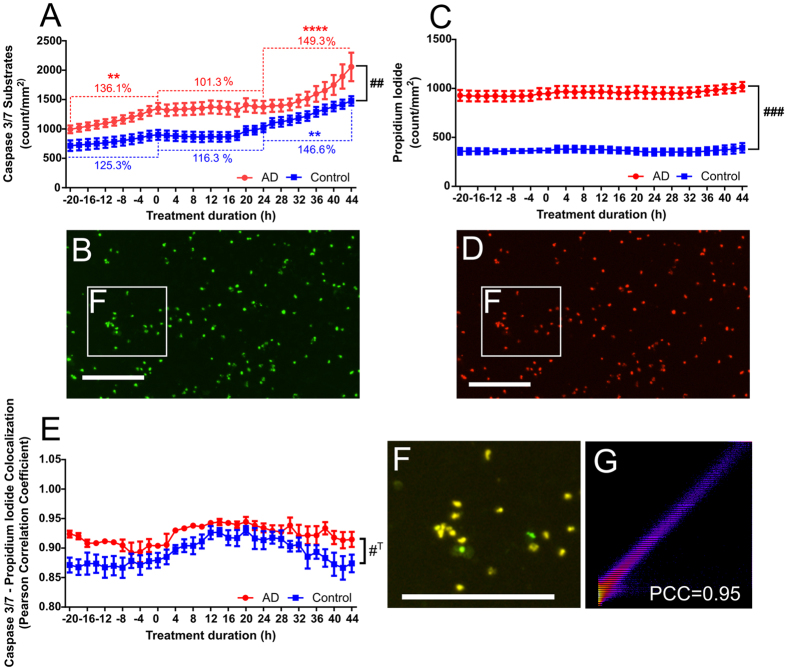
iPSC-derived neurons were generated from a sporadic AD patient or an age-matched control. Apoptosis and cell viability were simultaneously assessed after increasing oxygen from 3 to 19% 22 h prior to apigenin (10 μM) treatment. Apoptosis was assessed through quantifying caspase 3/7 substrates (**A**), number of substrates per mm^2^, n = 8) % of change between −22 h and 0 h, 0 h and 24 h and 24 h and 44 h indicated through dotted lines. Dead cells were quantified through counting of propidium iodide (**C**), number of cells per mm^2^, n = 4). Representative microscopy images of caspase 3/7 substrates (**B**) and propidium iodide (**D**) at 10×, scale = 200 μm. (**E**) Colocalization was determined through Pearson’s correlation coefficient for caspase 3/7 substrate signal with propidium iodide signal (1 = 100% colocalization, 0 = no colocalization). (**F**) Merged microscopy images of caspase 3/7 and propidium iodide signal at 10×, scale = 200 μm. (**G**) Representative 2D intensity histogram for green (y-axis, caspase 3/7 substrates) and red (x-axis, propidium iodide signal) pixels. Data presented as mean ± SEM; **p < 0.01, ***p < 0.001, ****p < 0.0001 as compared to % change between 0 h and 24 h time points; ^#T^p = 0.07, ^##^p < 0.01, ^###^p < 0.001 difference between AD and control cultures.

In order to further interrogate the potential neuroprotective effects of apigenin against oxidative stress, we differentiated iPSCs derived from a sporadic AD patient and an age and gender matched control into neurons with extended neurites in hypoxic conditions. After 75 days differentiation, neurons were transferred to atmospheric oxygen 24 h prior to apigenin (10 μM) treatment. AD and control neuronal cultures showed similar caspase 3/7 activity (993.7 vs 715.8 substrates/mm^2^ respectively, p > 0.9, Fig. 5A,B) at the beginning of the normoxic culturing period. Increasing O_2_ from 3 to 19% triggered a steady increase in caspase 3/7 substrates (AD: 136.1 ± 8.60%, Control: 125.3 ± 5.15%; Fig. 5A). Apigenin (10 μM) treatment slowed the production of caspase 3/7 substrates (AD: 101.3 ± 1.66%, Control: 116.3 ± 6.66% difference vs −22 h–0 h; Fig. 5A) for 24 h. The number of caspase 3/7 substrates significantly rose again for the following 24 h period (AD: 149.3 ± 13.28%, p < 0.001 vs 0 h–24 h; Control: 146.6 ± 6.1%, p < 0.01 vs 0 h–24 h; Fig. 5A), with more substrates produced by the AD neuronal cultures than control cultures (p < 0.01). Propidium iodide binding was not affected by the increase in O_2_ (Fig. 5C,D), with 2.62 ± 0.13 times more propidium iodide positive cells, as a marker of dead cells, in sporadic AD cultures than control cultures (1018.3 ± 40.9 vs 393.2 ± 39.3, respectively, p < 0.001; Fig. 5C). Caspase 3/7 substrates showed high colocalization with propidium iodide signal for sporadic AD and control neuronal cultures before and after apigenin treatment (Fig. 5E,G), with a trend for higher correlation in sporadic cultures than control cultures (p = 0.07, Fig. 5E,G). This data was further confirmed using Annexin V and propidium iodide (Supp. Fig. 4); together this data provided evidence for AD neuronal cell death via apoptosis in oxidative stress conditions, with protection from apoptosis afforded by apigenin.

### Apigenin reduces hyper-excitability in sporadic AD neurons

**Figure 6:**
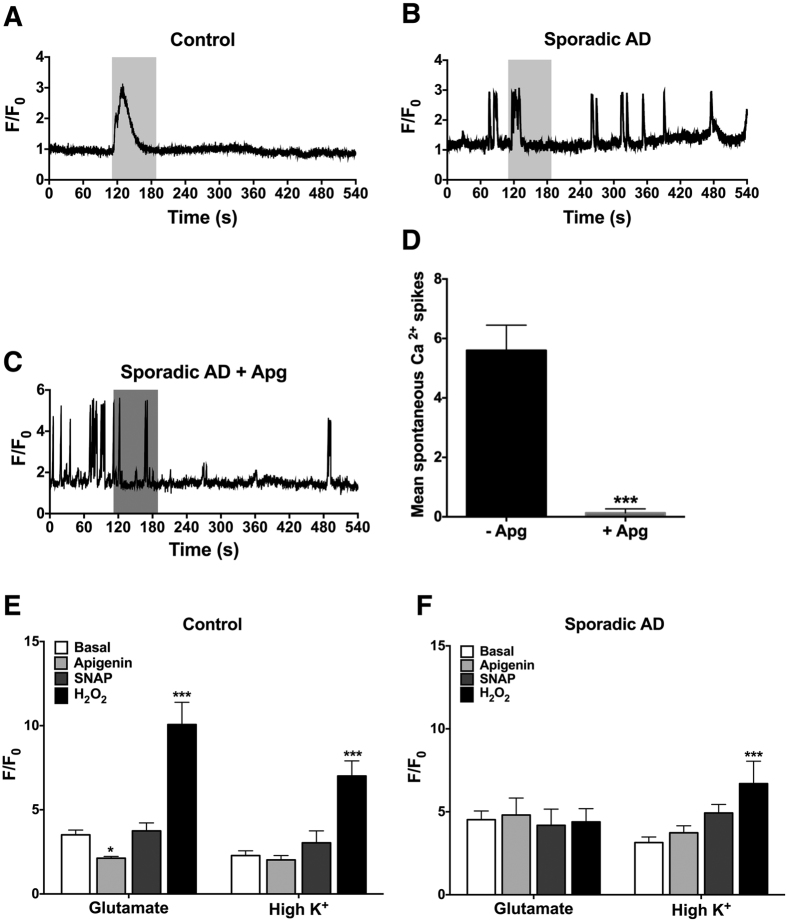
Representative images of Ca^2+^ imaging traces in (**A**) control, (**B**) sporadic AD derived neurons and (**C**) sporadic AD derived neurons exposed to apigenin. Neurons were loaded with fluorescent Ca^2+^ indicator Fluo3-AM and data are represented as mean change in fluorescence from baseline (F/F_0_) over time. Application of 60 mM KCl (high K^+^) or 50 μM apigenin is represented as the light or dark shaded area, respectively. Mean relative change in fluorescence was calculated from (**D**) control and (**E**) sporadic AD derived neurons. Loaded neurons were perfused with 50 μM apigenin, 300 μM H_2_O_2_, or 10 μM SNAP prior to 200 μM glutamate or 60 mM high K^+^. Data are presented as mean ± SEM of three independent experiments from >30 cells. Significant difference from basal response was calculated using two-way ANOVA with Tukey’s multiple comparisons test and is indicated by ***p ≤ 0.001.

We hypothesised that Ca^2+^ dysfunction, as a major disease phenotype accompanying AD^5^,^40^, could be contributing to the increased apoptosis observed in the sporadic AD neurons. Strikingly, through live cell Ca^2+^ imaging we observed that the sporadic AD neurons displayed a hyper-excitable phenotype, demonstrating multiple spontaneous Ca^2+^ responses in the absence of stimulus, whereas control neurons remained inactive in the absence of stimulus (Fig. 6A,B). Exposure to apigenin reduced the hyper-excitable phenotype observed in sporadic AD neurons, significantly reducing the frequency of spontaneous Ca^2+^ signalling (Fig. 6C,D).

Treatment with glutamate induced an influx of Ca^2+^ into both control and sporadic AD neurons (Fig. 6E,F). Exposure to apigenin (F(3, 159) = 31.05, p < 0.001) prior to glutamate significantly reduced the amplitude of the Ca^2+^ response in control neurons (P < 0.05) but not in sporadic AD neurons (Fig. 6E,F). Exposure of neurons to the NO donor, SNAP, prior to glutamate had no effect on the amplitude of the Ca^2+^ response in either control or sporadic AD neurons, while H_2_O_2_ significantly increased the glutamate induced Ca^2+^ response in control neurons only (p < 0.001).

Similar to glutamate, treatment with high K^+^ induced an influx of Ca^2+^ into both control and sporadic AD neurons. Exposure to apigenin or SNAP prior to high K^+^ had no effect on the amplitude of the Ca^2+^ response in either control or sporadic AD neurons. However, exposure to H_2_O_2_ prior to high K^+^ significantly increased the amplitude of the Ca^2+^ response in both control (p < 0.001) and sporadic AD neurons (p < 0.001).

Taken together, these data indicate a hyper-excitable phenotype in sporadic AD neurons that can be moderated by apigenin, and that apigenin is able to decrease glutamate-induced Ca^2+^ influx in healthy neurons.

## Discussion

Through the use of iPSC-based AD modelling, we performed a comprehensive analysis of the neuroprotective effects of apigenin against mediators of AD: inflammation, oxidative stress, disruption to neuronal Ca^2+^ signalling patterns, neurite retraction and apoptosis. For the first time in an iPSC model of AD we demonstrated a hyper-excitable phenotype in neuronal Ca^2+^ signalling. In addition, we also showed that AD neurons had a shorter neurite length, increased nitrite levels and increased cytotoxicity and apoptosis. Loss of neuronal viability was further enhanced in AD neurons following challenge with inflammatory or oxidative stress. Importantly, our results show that apigenin is able to protect these human neurons from neurite retraction and apoptosis, as well as reduce the hyper-excitable Ca^2+^ signalling phenotype.

### Neuroprotective potential of apigenin: anti-inflammatory activity

Patients who suffer from AD display higher levels of inflammation, oxidative and nitrosative stress, as evidenced by high levels of cytokines, chemokines and nitrated tyrosines present in patient brain tissue and cerebrospinal fluid^41^,^42^. As the development of AD is hypothesised to occur over several decades, this suggests that successful treatments need to be pre-emptive to prevent or delay disease onset, rather than reversing the pathogenic mechanisms once symptoms have developed, in addition to being suitable for long-term use. The dietary accessibility of plant-based polyphenolic compounds, like apigenin, makes them a suitable candidate as they have been shown to prevent microglial activation and may be potentially protective against AD progression^43^.

We first wanted to determine whether apigenin could protect against inflammatory-mediated neurite retraction, as one of the strongest pathogenic correlates with cognitive decline in AD is the loss of neurons from the retraction of neurites and neuronal death^39^. We demonstrated that iPSC-derived neurons generated from AD patients displayed a significant reduction in neurite length, which was exacerbated by exposure to media from activated inflammatory cells, in addition to elevated levels of nitrite, used as a downstream marker of NO. By using activated murine macrophage and microglial cells, our results indicate that apigenin has broad anti-inflammatory activity, which protects neurites by preventing activation of inflammatory pathways and reducing the release of NO. Potential mechanisms mediating neuroprotection by apigenin may involve the regulation of pro-inflammatory cytokines, such as IL-6, IL-1β and TNF-α by preventing NF-kB activation^20^.

The involvement of NO in the pathogenesis of AD is highly complex^35^. As our results, and reports from the literature suggest, activated glial cells can produce high concentrations of NO, which is generated by the enzyme inducible NO synthase (iNOS), while neurons produce much lower concentrations by the Ca^2+^ activated enzyme neuronal NO synthase (nNOS)^44^. When produced at low concentrations, NO plays an important role in synaptic plasticity and as a second messenger in the brain^45^. Indeed we found that low concentrations of NO protected neurons from apoptosis. However, at high concentrations NO can become neurotoxic, due to its propensity to react with the superoxide anion to form aggressive oxidants, such as peroxynitrite^46^. Studies using the N2a/Peuht39 neuroblastoma line have demonstrated that peroxynitrite can induce the nitration and aggregation of tau, which could potentially contribute to AD pathology^10^. Our results suggest that apigenin is able to protect neurons by reducing NO levels from activated glia, using a mechanism distinct from NO scavenging. However, our results also show that apigenin does not significantly reduce nitrite levels in neurons. Together, this indicates that the mechanism of action for apigenin may be specific to pathways associated with iNOS activation, as it was able to reduce nitrite levels in glial cells. The targeted action of apigenin towards iNOS generated NO is important in the physiological context of the brain as apigenin may reduce the potentially cytotoxic levels of NO produced by activated glia, without global scavenging of NO, allowing the lower concentrations of NO produced in neurons by nNOS (and in endothelial cells by eNOS) to continue mediating vital cellular functions and protection.

The suppressive effect of apigenin on iNOS activity has been recently reported in a diabetes-associated cognitive decline rat model^47^. This group also demonstrated that apigenin was able to reduce the activity of both nNOS and eNOS. Although we did not identify a reduction in nitrite in our cultures after apigenin treatment, under certain conditions the mechanism could involve reducing intracellular Ca^2+^, which is required for the function of calmodulin, an essential co-factor for nNOS and eNOS.

Understanding the action of apigenin on the different NOS enzymes is important due to the complex and often concentration dependent role of NO in the brain. High levels of reactive oxygen and nitrogen species mediating cytotoxic and apoptotic mechanisms in neuronal cell lines have previously been shown^48^,^49^. However low concentrations of NO, applied via a NO donor, were protective in our cells, significantly reducing apoptosis in both the AD and control neurons. In the brain, induction of the soluble guanylate cyclase and the cyclic guanosine monophosphate cGMP pathway by NO in neurons promotes vasodilation, increasing the cerebral blood supply to neurons and decreasing the potential for oxidative stress^50^,^51^. This complex and concentration dependent role of NO in the brain highlights the need to understand the specific action of apigenin in regards to inhibition of NO production by the different NOS enzymes and in different cell types.

The murine RAW264.7 macrophage cell line, along with the C8B4 microglial cell line, is often used as a model for inflammatory cells^18^. Activation of these cells with LPS and IFNγ results in the release of cytokines, chemokines and a synergistic and potent up-regulation of iNOS, leading to an increase in NO release^18^. For this reason RAW264.7 and C8B4 cells, along with primary microglia, were used to assess the anti-inflammatory properties of apigenin. However, an ideal model for understanding the effects of inflammatory activation on AD neurons would be to use microglia differentiated from iPSCs. Although there are protocols for generating microglia-like cells from mouse iPSCs^52^, these cells (and similarly for human iPSCs) do not release the comprehensive panel of cytokines and chemokines of primary microglia. An important future direction is therefore to develop human iPSC-derived microglia that can release the full complement of cytokines and chemokines upon activation.

Development of human iPSC-derived microglia is important as our data suggest that there are differences in the panel of cytokines and chemokines that are released from the different cell lines used in this study^53^. Activation of microglia is a relatively early pathogenic event in AD patients (prior to neuropil destruction), while transgenic AD mouse studies suggest that cytokines may also influence plaque deposition/degradation^54^. Pro-inflammatory mediators, such as TNFα and IL-1β, in combination with IFN-γ, can be directly cytotoxic to neurons when chronically produced at high concentrations^55^ and stimulate the production of Aβ peptides^56^. Conversely upon activation with Aβ, microglial cells produce the pro-inflammatory cytokines IL-1, IL-6 and TNFα and the chemokines MIP-1 and MCP-1^57^. This pathogenic and cyclical relationship between inflammatory cytokines and Aβ formation highlights the fundamental role of inflammation in AD and the need to develop human disease models to accurately understand the multiple and interconnected pathways.

Collectively, our results in the macrophage and microglial cells suggest that apigenin has broad anti-inflammatory activity, preventing activation of inflammatory pathways and NO release. Apigenin may therefore delay the onset or slow the progression of AD, targeting multiple pathogenic components associated with the early stages of disease progression by reducing the inflammatory response. Our data further suggests the activation of microglia could contribute to neurite retraction, synapse loss and neuronal death.

### Neuroprotective potential of apigenin: anti-apoptotic activity in neurons

The loss of neurons during AD is one of the strongest pathological correlates with cognitive decline, yet is a disease phenotype that has been difficult to recapitulate in animal models^28^,^58^. The AD neurons generated in this study demonstrated significantly higher levels of caspase-3/7 activity, two cysteine-aspartic proteases that play an essential role in apoptosis, highlighting the biological relevance of our iPSC-derived model of AD. Several groups have confirmed a significant increase in caspase-3/7 activity at the onset of cognitive decline during AD, closely correlating this change with synaptic loss and neuronal death^59^,^60^. Increases in caspase-3 and 7 activity were specifically associated with synaptic dysfunction in a mouse model of AD^60^, suggesting that therapeutic compounds aimed at protecting neurons against caspase-3/7 activation would be particularly beneficial against synaptic loss and cognitive decline.

We show that in a human iPSC-derived model of AD that apigenin is able to significantly reduce caspase-3/7 activity, protecting cells from apoptosis. Conversely, apigenin was unable to protect neurons from cytotoxicity, suggesting its mechanism of action may be caspase-specific. These findings are in accordance with previous studies showing that apigenin protects against caspase-3 induced apoptosis in SH-SY5Y cells by inhibiting the release of cytochrome *c* and thus preventing the activation of the caspase-3 cascade^25^.

The specific mechanisms triggering the observed increase of caspase-3/7 activity in AD neurons may be the result of AD mediated pathogenesis. Studies regarding the activation of caspase-3 in a number of AD transgenic mouse models have demonstrated that ROS, generated by toxic Aβ_1–42_ oligomers, can induce the release of cytochrome *c* from mitochondria, which then activates caspase-3^24^,^61^.

Together, these results demonstrate increased cytotoxicity and apoptosis in iPSC-derived sporadic AD neurons, which has been difficult to recapitulate in animal models of the disease. Furthermore, we showed that treatment with apigenin could significantly reduce caspase-3/7 meditated apoptosis in AD neurons, highlighting the potential therapeutic application of apigenin.

### Neuroprotective mechanism for apigenin: reducing neuronal hyper-excitability and Ca^2+^ dysfunction

Perturbed Ca^2+^ homeostasis may play a key role in AD pathogenesis, by stimulating the production of inflammatory cytokines, reactive nitrogen and oxygen species^62^ in neurons and glia, and as a central underlying mechanism in the activation of many downstream pathways^40^. Recently, a shift in the understanding of how Ca^2+^ dysfunction manifests and contributes to the functional impairment of neurons has begun to emerge. Increasing experimental evidence from mouse models and human patients with AD is suggesting that neuronal networks are hyperactive, especially in the early stages of disease progression^63^. In order to improve cell modelling of these complex phenotypes in neurodegenerative disorders, it is increasingly important to validate the differentiation of disease relevant cells from iPSCs by identifying and investigating disease phenotypes using functional assays (reviewed in ref. 64). The Ca^2+^ imaging experiments reported in this study demonstrate an endogenous hyper-excitability phenotype in iPSC-derived AD neurons. This provides a novel *in vitro* human model with which to investigate this emerging contributing factor of AD pathogenesis and a platform to test potential therapeutic compounds.

Imaging experiments on hippocampus CA1 neurons in double transgenic AD mice (APPswe/PS1M146V) have shown increased neuronal firing rates in association with reduced neurite length^65^. These results are in accordance with the AD neurons generated in this study, which had a hyper-excitable phenotype in the absence of stimulus and reduced dendrite length. In addition, although networks of hyperactive neurons have been found clustered around Aβ plaques, two-photon calcium imaging in young double transgenic mice (APP23/PS45) found populations of hyperactive CA1 neurons, prior to plaque formation, suggesting this is an early occurrence in AD pathogenesis^66^. The relatively short maturation time for the neurons generated in this study (*i.e.,* weeks, in comparison to years in the human brain), in conjunction with the elevated levels of secreted Aβ_42_ could act as a proxy for earlier stages of AD progression. Our results also suggest that culturing neurons at atmospheric O_2_ promotes oxidative stress that is able to drive pathogenesis and disease phenotype in the cells. A potential mechanism driving the hyper-excitable phenotype is that elevated levels of Aβ, as measured in the AD neurons, have the capacity to modify receptors involved in Ca^2+^ signalling, such as NMDA receptors, increasing intracellular Ca^2+^ and altering signalling patterns^40^.

Exposure to apigenin reduced the frequency (but not amplitude) of Ca^2+^ signals in the AD neurons. This is the first report of apigenin modulating hyper-excitability in a model of AD, demonstrating a further neuroprotective mechanism against AD pathogenesis. Intriguingly, apigenin reduced the mean Ca^2+^ response to glutamate in control neurons but not in AD neurons; the loss of this regulatory mechanism in AD neurons remains unidentified and will be the subject of future research. There are conflicting reports in the literature regarding the effect of apigenin on receptors involved in Ca^2+^ signalling. In cultured rat cortical neurons apigenin has been shown to decrease NMDA receptor function, with no effect on AMPA receptor activity^26^, however in cultured rat hippocampal neurons, apigenin was shown to inhibit both NMDA and AMPA receptors^67^. Identifying the specific effect of apigenin on these important signalling channels in human neurons should be investigated to better understand the protective mechanism of apigenin.

The iPSC-derived neurons used in this study express NMDA receptor subunits (data not shown), indicating that the observed effects in these human cells could be mediated via NMDA receptors. There are a number of potential mechanisms facilitating the observed reduction in neuronal hyper-excitability by apigenin. The reported anti-oxidative and anti-amyloidogenic properties of apigenin in transgenic APP mouse models of AD^23^, in conjunction with the ability of apigenin to reduce NO production, may protect against oxidative modification of NMDA receptors, as a result of reactive oxygen species generated by Aβ_42_. In addition, apigenin protection could be mediated through inhibition of protein kinase C (PKC) phosphorylation of the NMDA receptor. Phosphorylation of the core NR1 subunit of the NMDA receptor potentiates Ca^2+^ influx through the receptor^68^. Studies have shown that the polyphenolic compound, curcumin, is able to inhibit PKC phosphorylation of NMDA receptors in primary rat cerebral cortex cells^68^, and that apigenin and curcumin can inhibit PKC activity in NIH3T3 cells^69^. However, it has yet to be confirmed if apigenin alters PKC activity in neurons and should be the focus of future studies.

The decreased glutamate-induced Ca^2+^ response to apigenin suggest a potential protective effect through a reduction in Ca^2+^ signalling, with the associated benefits of reduced Ca^2+^ signalling widely reported for AD (reviewed in ref. 70). For example, NMDA receptor antagonists, such as Memantine, are among the limited pharmacological compounds currently available for AD^71^. Attenuation of increased Ca^2+^ signalling may reduce some of the widespread downstream consequences, such as the activation of glial cells, the production of inflammatory cytokines and even a reduction in the formation of toxic Aβ oligomers, which can ultimately lead to neuronal death^72^.

The neuronal hyper-excitability observed in the AD neurons used in this study was also accompanied by significantly elevated levels of nitrite, used as a downstream marker of NO production. This suggested that the iPSC-derived AD neurons had a higher level of basal NO production. In post-synaptic neurons, the generation of NO is Ca^2+^ dependent, with nNOS co-localised with glutamate activated NMDA receptors^73^. The hyper-excitability in the AD neurons could be contributing to the elevated levels of nitrite. A recent study using the triple transgenic AD mouse model (3xTg-AD) suggested that elevated levels of NO may be a compensatory mechanism employed by neurons to boost synaptic transmission in the early stages of AD^74^. However, the sustained generation of NO due to neuronal hyper-excitability could lead to the formation of damaging reactive nitrogen and oxygen species, resulting in a pathogenic shift from a neuroprotective mechanism to a mediator of neurodegeneration. The hyper-excitable phenotype and elevated levels of nitrite present in this iPSC-derived model of AD provide a disease and species-relevant platform to investigate and confirm this proposed mechanism.

## Conclusion

Collectively, we report a hyper-excitable Ca^2+^ signalling phenotype in an iPSC-derived model of AD, as well as significantly elevated levels of apoptosis and nitrite, in association with decreased neurite length. We showed that treatment of AD neurons with apigenin reduces neuronal hyper-excitability and apoptosis. Furthermore, through the use of activated inflammatory cells we demonstrated that apigenin could inhibit the activation of cytokines and NO production, protecting AD neurons from inflammatory induced stress and neurite retraction.

Human iPSC-derived neuronal models provide opportunities to understand the neuronal functions and mechanisms of disease. Findings from our study suggest that the flavonoid apigenin has broad neuroprotective effects against a suite of pathogenic factors mediating AD progression, highlighting its potential as a therapeutic agent.

## Materials and Methods

### Derivation of control and AD-specific iPSCs

Skin samples were collected from patients and healthy individuals (all male), including: a 39-year old early-onset familial AD patient (PSEN1^P117R^; APOE3/3); a 29-year old healthy individual (APOE4/4); a 65-year old late-onset sporadic AD patient (APOE3/4); a 57-year old healthy individual (APOE3/3). All experimental protocols were approved by the University of Wollongong and University of New South Wales Human Research Ethics Committees (HE13/299 and HREC08037, respectively). The methods were carried out in accordance with the guidelines as set out in the National Statement on Ethical Conduct in Research Involving Humans and informed consent was obtained from all donors. Generation of human feeder-free iPSCs was carried out as previously described (Chung *et al.*, 2012). The iPSCs were considered pluripotent by teratoma assay and were karyotyped to confirm the lack of chromosomal changes during reprogramming. The iPSCs were cultured on Matrigel (Corning) coated tissue culture plates in mTeSR1 (Stem Cell Technologies) containing 25 U/mL Penicillin-streptomycin at 37 °C, 5% CO_2_ in a humidified incubator.

### Differentiation of control and AD-specific iPSCs to neurons

Differentiation of iPSCs was performed via neurospheres as per^75^. On day 29, neurospheres were dissociated with accutase, re-suspended in neural expansion media (DMEM/F12) supplemented with N2, B-27, Minimum Essential Medium (MEM) nonessential amino acids and heparin (1 mg/mL)) and triturated into single cells. Cells were plated out on laminin-coated 18 mm glass coverslips for Ca^2+^ imaging or in 24 well tissue culture plates for assays in neural expansion media at a density of 25,000 cells / well.

All growth factors were from R&D systems. Following overnight incubation, media was aspirated and replaced with media supplemented with 100 ng/mL of sonic hedgehog (SHH) and fibroblast growth factor 8 (FGF-8). On day 36, media was supplemented with 200 μM ascorbic acid in addition to SHH and FGF-8. On day 44 media was supplemented with 100 ng/mL each of cyclic adenosine monophosphate (cAMP), brain-derived neurotrophic factor, glial-derived neurotrophic factor and insulin-like growth factor 1, in addition to SHH, FGF-8 and ascorbic acid until day 75. Partial media changes were performed every other day for the entire culture period. For inflammation-based assays, neurons were treated with 50 μM apigenin or vehicle for 24 h, prior to complete media changes with conditioned media from activated microglia for 48 h. For all other assays neurons were treated with apigenin 50 μM or vehicle for 24 h, prior to complete media changes containing either 10 μM SNAP or 300 μM hydrogen peroxide (H_2_O_2_) for 24 h.

### Immunocytochemistry

Samples of iPSCs and neurons were stained for specific markers to confirm pluripotency and differentiation into mature neurons, respectively. Both iPSCs and neurons were plated on poly-L lysine (BD Biosciences) laminin-coated 8 mm coverslips. Cells were fixed with 4% (v/v) paraformaldehyde, permeabilised with 0.5% (v/v) TritonX-100 in phosphate buffered saline (PBS) and blocked with 5% (w/v) bovine serum albumin in PBS.

The iPSC colonies were stained with Oct3/4 (mouse 1:500, Stem Cell Technologies) primary antibody and anti-mouse Alexa Fluor 488 (1:1000, Life Technologies) secondary antibody. Neurons were stained with primary antibodies to microtubule-associated protein 2 (MAP2, 1:500), glial fibrillary acidic protein (GFAP, 1:250), synapsin-1 (SYN-1, 1:500) and postsynaptic density protein 95 (PSD-95, 1:500). Antibodies were from Millipore unless otherwise stated and were incubated with cells in blocking solution overnight at 4 °C. Anti-mouse/anti-rabbit isotype controls (Life Technologies) were used as negative controls. Anti-rabbit Alexa Fluor 488 and anti-mouse Alexa Fluor 633 secondary antibodies were used at 1:1000 in blocking solution for 1 h at room temperature. Cells were mounted in SlowFade Gold antifade reagent with DAPI (Life Technologies). Immunocytochemical preparations were imaged with a Leica DMI6000B confocal microscope and acquired using LAS AF 2.6 (Leica microsystems).

### Primary microglia isolation and cell line culture

RAW264.7 and C8B4 cells were maintained in Dulbecco’s Modified Eagle’s Medium (DMEM) supplemented with 200 U/ml penicillin, 200 μg/ml streptomycin and 10% foetal bovine serum (FBS) and incubated at 37 °C in 5% CO_2_. For primary microglial culture, brains were removed from murine E18 embryos from C57/Bl6 mice and meninges were removed. Cell cultures were washed twice with phosphate buffered saline (PBS; pH 7.2) followed by a 5 min incubation with 2 ml of 0.05% TrypLE Express (Life technologies) at 37 °C. Dislodged cells were resuspended in 10 ml of ice-cold separation buffer (PBS, pH 7.2, 0.5% bovine serum albumin (BSA) and 2 mM EDTA). The buffer was degassed in an ultrasound water bath (Unisonics, Brookvale, Australia), to prevent air bubbles from blocking the column. Cells were centrifuged at 300 g for 10 min and the supernatant discarded. Up to 1 × 10^7^ cells were resuspended in 90 μl of separation buffer (Miltenyi Biotec). The cells were incubated with 20 μl CD11b magnetic beads on a MS column (Miltenyi Biotec) for 15 min at 4 °C, washed with 2 ml separation buffer and centrifuged at 300 g for 10 min. The supernatant was removed completely and the cells were resuspended in 500 μl of separation buffer. Cells were passed through a 40 μm nylon mesh to remove cell clumps and plated into a T25 flask.

### Activation of microglia with LPS and IFN-γ and treatment with apigenin

Immediately after CD11b magnetic bead isolation primary microglia cells were seeded into 96 well Primaria tissue culture plates (Bio-Rad) at a density of 3 × 10^5^ cells per well. The conditioned media from the mixed culture was diluted 1:2 with fresh medium (total volume in each well 100 μl). Primary microglia, RAW264.7 and C8B4 cells were plated 24 h before incubation with apigenin for 1 h. Lipopolysaccharide (LPS; 50 μg/ml) and Interferon-γ (IFN-γ; 20 U/ml) were added, followed by incubation for 48 h. The supernatant of three wells was combined, spin filtered (0.22 μm Corning Costar Spin-X spin filter with a cellulose acetate membrane) and 0.4 μl protease inhibitor cocktail containing Aprotinin, Bestatin, E-64, Leupeptin and Pepstatin A was added. Of the supernatant 50 μl were immediately used for Griess assay and the remainder was stored at −80 °C for the cytokine assay.

### Resazurin-based assay for determination of cell viability

Cell viability of activated inflammatory cells or iPSC-derived neurons was assessed in terms of the metabolic capability of cells to convert the non-fluorescent redox indicator, resazurin, into its highly fluorescent product, resorufin. Briefly, 100 μl DMEM containing 0.125 mg/l resazurin (Sigma-Aldrich) was added to each well of a 96-well plate and incubated for 90 min at 37 °C. Fluorescence was measured at 560 nm excitation / 590 nm emission using a POLARstar Omega fluorescent plate reader (BMG LABTECH).

### Nitrite determination by Griess assay

The nitrite concentration in the culture medium of activated inflammatory cells or iPSC-derived neurons was used as an indicator of NO production using the Griess reaction. Cell culture medium in the absence of cells was also assessed to investigate the scavenging action of apigenin, with fresh media exposed to SNAP (0, 1, 10, 100, 1000 μM) and apigenin (0, 5, 10, 50 μM). The supernatant (50 μl from each well) was transferred to a fresh 96 well plate with 50 μl Griess reagent (25 μl of 1% sulphanilamide and 25 μl of 0.1% napthyethylene-diamine in 5% HCl). After 10 min incubation at room temperature, the absorbance of each well was measured at 540 nm. Nitrite concentration was calculated with reference to a standard curve of sodium nitrite generated by known concentrations, ranging from 0-100 μM.

### Neuron and neurite length measurements

Analyses were performed with High Content Analysis software HCA-Vision (CSIRO) to assess the neuronal images: Neuron Body Detection, Neurite Detection and Neurite Analysis. Images were background corrected and analysis was performed as in ref. 76.

### Apoptosis and cytotoxicity assay

The ApoTox-Glo Triplex assay (Promega) was performed according to the manufacturer’s guidelines to assess apoptosis, cytotoxicity and cell viability in parallel. Differentiated iPSC-derived neurons were pre-treated in the presence or absence of apigenin (10 μM) for 24 h at 37 °C and 5% CO_2_. Neurons were exposed to vehicle control, H_2_O_2_ (300 μM) or SNAP (10 μM) for 24 h at 37 °C and 5% CO_2_. All measurements were taken after 30 min incubation using a Polarstar Omega plate reader (BMG Labtech).

### Apoptosis and cytotoxicity assay over time

Differentiated iPSC-derived neurons were transferred to normoxic (37 °C, 5% CO_2_ and atmospheric O_2_) conditions after differentiation in hypoxic (3% O_2_) conditions. The culture media was supplemented with caspase 3/7 NucView 488 enzyme substrate (2.5 μM final concentration, Biotium #10402) and either CF594 Annexin V (2.5 μM final concentration, Biotium #30067) or propidium iodide (1 μg/ml final concentration, Sigma #P4884) 24 h prior to apigenin treatment (10 μM). Phase contrast and fluorescent images were captured using an IncuCyte Zoom (Essen Bioscience) at 2 h intervals and 10× magnification. Caspase substrates, propidium iodide-positive cells and annexin V confluence were quantified using the in-built basic analyser algorithm (Table 1) from a minimum of 8 images per well and time point after removing green (8%) and red (5%) channel bleed through. The colocalization of caspase 3/7 substrates with propidium iodide was analysed using the Coloc 2 ImageJ plugin (NIH). Pearson’s correlation coefficient and Manders split coefficients were used to determine colocalization of caspase 3/7 substrates with propidium iodide signal.

### Calcium imaging

To confirm the differentiation of functional neurons from iPSCs and compare differences in Ca^2+^ responses between iPSC derived neurons following treatments with acute application of apigenin, SNAP, H_2_O_2_ and glutamate, live cell fluorescence Ca^2+^ imaging experiments were performed. Images were captured at a rate of 1 frame per 0.112 s using a Leica confocal SP5 microscope and excitation at 488 nm with a 10× objective.

Neurons (day 75) were loaded with 3 μM Fluo3-AM (Life Technologies) and 0.04 μM pluronic acid (Biotium) in standard bath solution (SBS), containing 160 mM NaCl, 2.5 mM KCl, 5 mM CaCl_2_, 1 mM MgCl_2_, 10 mM HEPES, and 5 mM glucose, pH 7.4, for 15 min at 37 °C in the dark. Neurons were washed for 5 min in SBS at room temperature in the dark.

Neurons were perfused with 5 mL SBS at 2.2 mL/min using a Scintilla EpiPump to attain a baseline reading of fluorescence intensity (F_0_). Treatment regimes included 50 μM apigenin, 10 μM SNAP or 300 μM H_2_O_2_ prior to 200 μM glutamate and 60 mM KCl. Cells were considered for further analysis if they responded to 60 mM KCl (90% of cells responded to KCl, and there was no significant difference in the % responders among control or AD neurons). During post imaging analysis more than 30 individual cells were selected as regions of interest (ROIs) from three independent differentiation experiments, with fluorescence data background subtracted. Evoked Ca^2+^ responses for ROIs were expressed as the relative change in fluorescence intensity (F) to the baseline fluorescence signal of the cell (ΔF/F_0_).

### Statistical analysis

Paired T-test, one-way and two-way ANOVAs, as appropriate, were used to analyse the effects and interactions between treatments, followed by Tukey’s multiple comparisons test, results were considered significantly different at p < 0.05.

## Additional Information

**How to cite this article**: Balez, R. *et al.* Neuroprotective effects of apigenin against inflammation, neuronal excitability and apoptosis in an induced pluripotent stem cell model of Alzheimer’s disease. *Sci. Rep.* **6**, 31450; doi: 10.1038/srep31450 (2016).

## Supplementary Material

Supplementary Information

## Figures and Tables

**Table 1:** Mask parameters for Incucyte Basic analyser image analysis.

Targets	Channel	Exposure (ms)	Background Correction	Edge Sensitivity	Minimum Particle Size (μm^2^)	Maximum Particle Size (μm^2^)	Data presentation
Caspase 3/7 Substrates	Green	400	Top-Hat (10 μm, 2 GCU)	0	10	∞	Counts/mm2
Propidium Iodide	Red	800	Adaptive (2 RCU)	0	∞	∞	Counts/mm2
Annexin V	Red	800	Top-Hat (20 μm, 1 RCU)	−27	∞	∞	% Confluence
