# Supplementary material for: Neuroprotective effects of apigenin against inflammation, neuronal excitability and apoptosis in an induced pluripotent stem cell model of Alzheimer’s disease
Source: Sci Rep. 2016 Aug 12;6:31450. doi: 10.1038/srep31450 (PMC4981845; doi:10.1038/srep31450)
Supplement: Supplementary Information [file srep31450-s1.doc]

**Supplementary Information**

Neuroprotective effects of apigenin against inflammation, neuronal excitability and apoptosis in an induced pluripotent stem cell model of Alzheimer’s disease

Rachelle Balez, Nicole Steiner, Martin Engel, Sonia Sanz Muñoz, Jeremy Stephen Lum, Yizhen Wu, Dadong Wang, Pascal Vallotton, Perminder Sachdev, Michael O’Connor, Kuldip Sidhu, Gerald Münch and Lezanne Ooi.

SUPPLEMENTARY TABLES

Supplementary Table 1

Effect of apigenin on pro-inflammatory cytokine levels in RAW264.7, C8B4 and primary microglia (PM). A panel of 23 cytokines was measured, using a multiplex sandwich ELISA, according to the manufacturer’s protocol (Bio-Rad). Nitrite concentration was measured by Griess assay; effect of apigenin on nitrite levels: RAW264.7 IC_50_ 48±7 µM; C8B4 IC_50_ 50±9 µM; PM IC_50_ 30±6 µM.

| Cytokine | Cells | Apigenin IC_50_ (µM) |
| --- | --- | --- |
| IL-1α | RAW264.7 | 74±13 |
|  | C8B4 | 42±6 |
|  | PM | 14±8 |
| IL-1β | RAW264.7 | >75 |
|  | C8B4 | 67±10 |
|  | PM | 14±6 |
| IL-6 | RAW264.7 | 46±143 |
|  | C8B4 | 63±6 |
|  | PM | 38±9 |
| IL-9 | RAW264.7 | >75 |
|  | C8B4 | 55±22 |
|  | PM | >75 |
| IL-12(p40) | RAW264.7 | >75 |
|  | C8B4 | 73±12 |
|  | PM | 37±24 |
| IL-12(p70) | RAW264.7 | 70±5 |
|  | C8B4 | 72±12 |
|  | PM | >75 |
| TNF-α | RAW264.7 | 75±9 |
|  | C8B4 | 27±3 |
|  | PM | 19±18 |
| G-CSF | RAW264.7 | >75 |
|  | C8B4 | 73±7 |
|  | PM | >75 |
| GM-CSF | RAW264.7 | >75 |
|  | C8B4 | >75 |
|  | PM | >75 |

Supplementary Table 2

Effect of apigenin on anti-inflammatory cytokine levels in RAW264.7, C8B4 and primary microglia (PM), measured by cytokine array (Bio-Rad).

| Cytokine | Cells | Apigenin IC_50_ (µM) |
| --- | --- | --- |
| IL-10 | RAW264.7 | 48±7 |
|  | C8B4 | 57±15 |
|  | PM | 49±2 |
| IL-13 | RAW264.7 | >75 |
|  | C8B4 | >75 |
|  | PM | >75 |

**Supplementary Table 3**

Effect of apigenin on chemokine levels in RAW264.7, C8B4 and primary microglia (PM), measured by cytokine array (Bio-Rad).

| Cytokine | Cells | Apigenin IC_50_ (µM) |  |
| --- | --- | --- | --- |
| Eotaxin | RAW264.7 | >75 | |
|  | C8B4 | >75 | |
|  | PM | >75 | |
| KC | RAW264.7 | >75 | |
|  | C8B4 | >75 | |
|  | PM | 67±64 | |
| MCP-1 | RAW264.7 | 49±8 | |
|  | C8B4 | >75 | |
|  | PM | 28±4 | |
| Mip-1α | RAW264.7 | >75 | |
|  | C8B4 | >75 | |
|  | PM | 11±33 | |
| Mip-1β | RAW264.7 | 27±7 | |
|  | C8B4 | >75 | |
|  | PM | >75 | |
| RANTES | RAW264.7 | >75 | |
|  | C8B4 | >75 | |
|  | PM | >75 | |

**SUPPLEMENTARY FIGURES**

**Supplementary Figure 1**

Neurons were harvested at day 35 of differentiation for immunoblot analysis.

For immunoblot analysis, cells were washed with phosphate buffered saline and lysed with

Cell Signalling Lysis Buffer (Millipore). Cells were then centrifuged at 3000 x *g* for 5

minutes and supernatant was collected for immunoblot analysis. Protein (10 ug) was resolved

in Criterion XT Bris-Tris Precast Gels (BioRad) and transferred onto Immuno-Blot PDVF

membrane (BioRad). Membranes were blocked with 5% Bovine Serum Albumin (BSA) for 1

hour at room temperature. Primary antibodies were incubated overnight at 4 oC to identify

proteins of interest: anti-PSD-95 (Millipore MAB1598, 1:1000), anti-synapsin1 (Millipore

MAB1543, 1:1000), anti-MAP2C (Sigma M4403), anti-actin (Millipore MAB1501, 1:50

000). All antibodies were subsequently incubated with HRP-conjugated secondary

antibodies. Blots were visualised with chemiluminesence western blot detection kit

(Amersham) and developed using the AGFA CP1000 film developer (Agfa-Gevaert N.V.,

Mortsel, Belgium).

**Supplementary Figure 2**

Quantitation of (A) Aβ_40_ and (B) Aβ_42_ peptides by ELISA (life technologies) from healthy individual (control) or sporadic AD neurons. Data shown are mean ± SEM, n=3, *p<0.05 t-test.


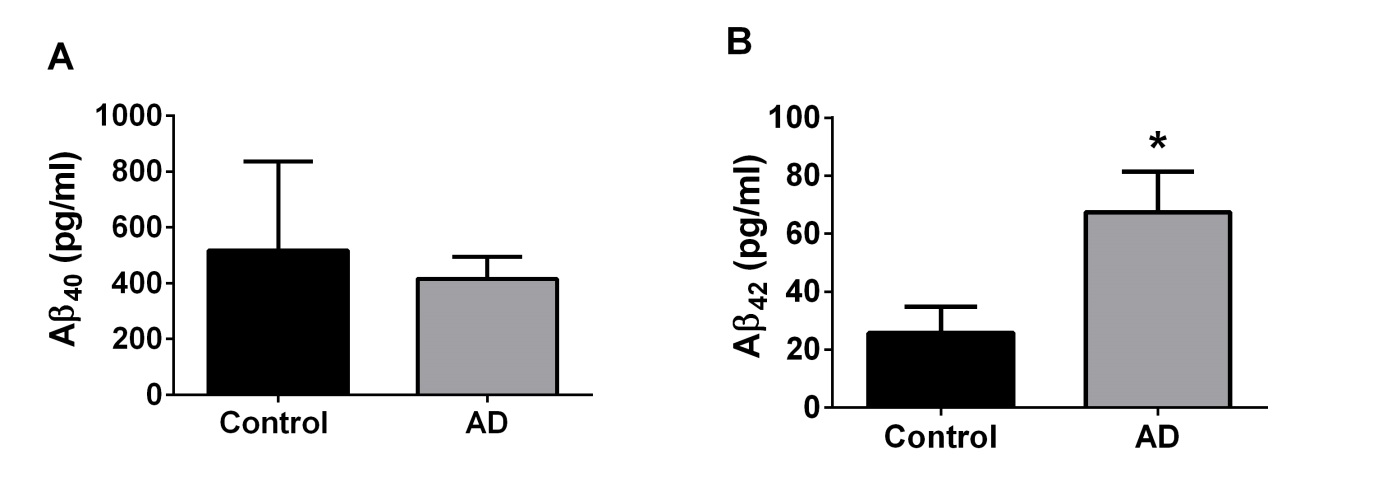


**Supplementary Figure 3**

Representative western blots and quantification of neuronal, axonal, dendritic and synaptic markers in sporadic AD and control neurons after 52 days of differentiation.

Control and sAD neurons were harvested at day 52 for protein quantification by western blot analysis. Neurons were washed with phosphate buffered saline and lysed with RIPA buffer containing protease and phosphatase inhibitors. Cells were then centrifuged at 10,000 g for 5 minutes and the supernatant collected. Protein (5 ug) was resolved in Criterion TGX-Precast Gels 4-20% gels (BioRad) and transferred onto GE-Life Sciences PDVF membrane. Membranes were blocked with 5% BSA or 5% milk (MAP-2C), for 1 hour at room temperature. Primary antibodies were incubated overnight at 4°C to identify proteins of interest: anti-NeuN (Abcam ab177487, 1:2,500), anti-NSE (Abcam ab53025, 1:10,000), anti-synapsin-1 (Millipore ab1543, 1:5,000), anti-neurofilament heavy chain (NF-HC) (Abcam ab8135, 1:10,000), anti-MAP2C (Millipore MAB3418, 1:15,000) and anti-GAPDH (Osenses OSG00032W, 1:10,000). All antibodies were subsequently incubated with HRP-conjugated secondary antibodies. Blots were visualised with chemiluminesence western blot detection kit (Amersham) and developed using the AGFA CP1000 film developer (Agfa-Gevaert N.V., Mortsel, Belgium).

Representative western blots and quantification are shown for neuronal specific markers NeuN and neuron specific enolase (NSE), axonal and dendritic markers neurofilament-heavy chain (NF-HC) and microtubual associated protein-2C (MAP-2C), respectively, and synaptic marker Synapsin-1 (Syn-1) expression was normalised to GAPDH. Protein data are presented as mean ± SEM, n=6, *p<0.05, t-test.

**
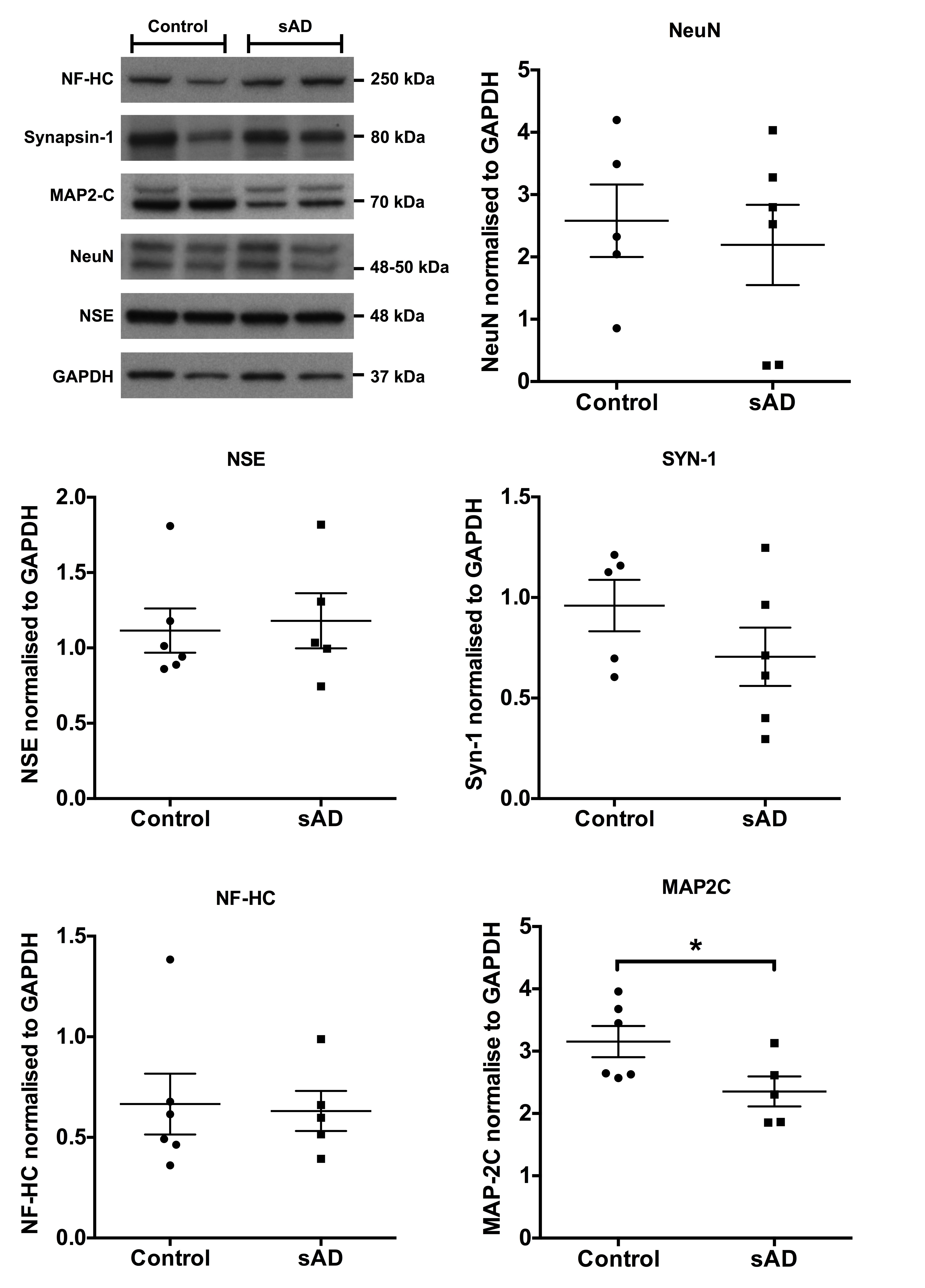
**

**Supplementary Figure 4**

**Annexin V counts confirm caspase 3 activity results**

Live cell imaging experiments were used to supplement the caspase 3/7 activity results in Figures 4 and 5, by assessing the number of Annexin V positive cells (labelled with AnnexinV-FITC; Biolegend #640906; 2.5 µl for 400,000 cells) in relation to propidium iodide positive cells (Sigma-Aldrich #P4864; 10 µg/ml for 400,000 cells) following apigenin treatment (10 uM Apg) or vehicle control treatment (Veh) after increasing oxygen from 3 to 19%. Cells were imaged in an Incucyte every 24 h and Annexin V positive cells were counted in iPSC-derived neuronal cultures from sporadic Alzheimer's disease patient (sAD) and healthy control cells (Con). Untreated AD cultures showed a significant increase in Annexin V levels (* p<0.05 vs APG), while apigenin treated AD cultures did not differ from control cultures. To control for the possible change in Annexin V levels of already apoptotic cells, we assessed the propidium iodide count, which did not change over the same period, suggesting that the increase in Annexin V count is the result of formerly healthy cells undergoing apoptosis. The Pearson correlation coefficients for Annexin V / PI were Control – Vehicle 0.94; Control – Apg 0.95; sAD - Vehicle 0.83; sAD – Apg 0.90.

**
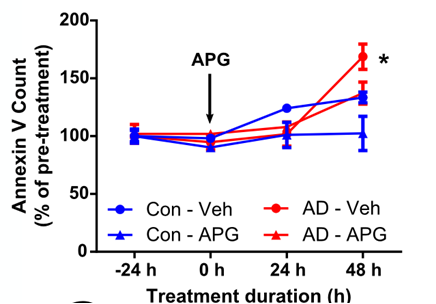
**
